# Structural and functional analysis of *Bacillus* sarcosine oxidase and its activity toward cyclic imino acids

**DOI:** 10.1002/2211-5463.70119

**Published:** 2025-09-11

**Authors:** Yuqi Zhang, Yoshitaka Nakajima, Masae Kurobe, Tsutomu Nakamura, Tomoki Himiyama, Yoshiaki Nishiya

**Affiliations:** ^1^ Division of Life Science, Graduate School of Science and Engineering Setsunan University Osaka Japan; ^2^ Biomedical Research Institute National Institute of Advanced Industrial Science and Technology Osaka Japan

**Keywords:** *Bacillus* sp., crystallization, cyclic imino acids, FAD, mutagenesis, sarcosine oxidase

## Abstract

This study investigated the reactivity of sarcosine oxidase (Sox) toward minor substrates through kinetic and structural analyses, along with mutational engineering to elucidate their reaction mechanisms. Sarcosine oxidase from *Bacillus* sp. (SoxB) recognizes the cyclic imino acids l‐proline (l‐Pro), d‐proline (d‐Pro), and l‐thioproline (l‐Tpr) as minor substrates. The reaction behavior varied depending on the substrates; notably, the absorption spectrum of l‐Tpr exhibited charge transfer, which was characteristic of substrate inhibition. Crystal structures of the enzyme–substrate complexes suggested that Tyr254 causes spatial interference with cyclic imino acids at the active site. The Tyr254Ala and Tyr254Gly mutants exhibited enhanced reactivity toward cyclic imino acids by eliminating this spatial interference. Crystallographic analysis of the mutants revealed an enlarged active site, which facilitated reactions with five‐membered cyclic imino acids. These mutations disrupted the electron delocalization associated with l‐Tpr, thereby eliminating charge transfer and substrate inhibition. A water network was also identified near the enzyme's active site, interacting with the side chain of Tyr254. These findings provide valuable insights into substrate specificity and may facilitate the development of enzymes with broader substrate scope and enhanced catalytic activity.

## Graphical abstract

Sarcosine oxidase from *Bacillus* sp. (SoxB) recognizes l‐proline (l‐Pro), d‐proline (d‐Pro), and l‐thioproline (l‐Tpr) as minor substrates. In this study, we measured their absorption spectra, determined the crystal structures of their enzyme complexes, and performed site‐directed mutagenesis of key residues. These approaches successfully enhanced the enzyme's activity toward the cyclic imino acids. The findings provide valuable insights for improving enzyme performance.

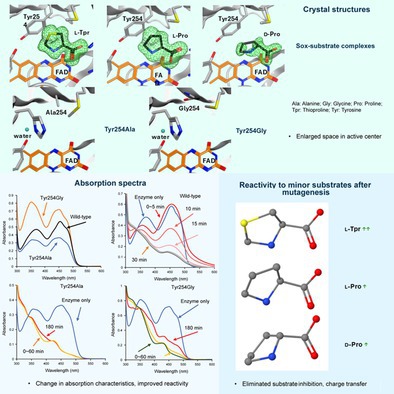

**Abbreviations**
DMG: *n*‐dimethylglycine
d‐Pro: d‐proline
FAD: flavin adenine dinucleotide
FAD^ox^
: enzyme's oxidized form of FAD
FAD^red^
: enzyme's reduced form of FAD
HACET: hydrogen‐atom‐coupled electron transfer
l‐Pro: l‐proline
PDB: Protein Data Bank
SDS/PAGE: sodium dodecyl sulfate/polyacrylamide gel electrophoresis
Sox: sarcosine oxidase
SoxA: Sox from the *Arthrobacter* sp
SoxB: Sox from the *Bacillus* sp
Tpr: thiazolidine‐4α‐carboxylic acid (thioproline)

Enzymes in living organisms generally exhibit high substrate specificity; however, non‐native substrates typically show weak reactivity [1, 2]. For example, enzymes such as lactate dehydrogenase (LDH) and malate dehydrogenase (MDH), as well as monomeric and heterotetrameric sarcosine oxidases exhibit this property.

Monomeric sarcosine oxidase (Sox, EC:1.5.3.1) is an oxidoreductase that contains flavin adenine dinucleotide (FAD) as a coenzyme and primarily catalyzes the demethylation of sarcosine (*n*‐methylglycine). Due to structural similarities, Sox family enzymes are commonly classified as d‐amino acid oxidase‐like enzymes (SCOP ID: 4000124). However, notable differences exist among Sox and related enzymes within this family [3]. Because heterotetrameric sarcosine oxidase reacts exclusively with l‐substrates [4], its stereoselectivity is generally considered l‐specific [5, 6]. Nevertheless, Sox has been reported to react with d‐substrates, although the detailed mechanism remains unclear [7].

The Sox‐catalyzed reaction comprises two half‐reactions: a reductive half‐reaction, in which the substrate transfers a hydrogen atom to the oxidized form of FAD (FAD^ox^), forming an intermediate and the reduced form (FAD^red^); and an oxidative half‐reaction, in which FAD^red^ donates a hydrogen atom to molecular oxygen, producing hydrogen peroxide and regenerating FAD^ox^ [8]. Fragment molecular orbital analyses based on X‐ray crystal structures, along with combined quantum and molecular mechanical calculations, have revealed that the reductive half‐reaction of Sox occurs primarily through hydrogen‐atom‐coupled electron transfer (HACET). In this mechanism, one hydrogen atom is transferred from the methyl group of sarcosine to the N5 atom of the isoalloxazine ring of FAD, while an electron is concurrently transferred from the nitrogen atom of sarcosine to the C4x atom of the isoalloxazine ring [9]. However, the reaction with *n*‐cyclopropylglycine does not proceed via the HACET mechanism. Instead, it follows a polar mechanism [10, 11], in which the nitrogen atom of the substrate first binds to the carbon atom of FAD, followed by electron transfer [12]. Thus, the reaction mechanism of the same enzyme can vary depending on the substrate.

Sox exhibits reactivity not only toward sarcosine and *N*‐cyclopropylglycine but also shows low reactivity toward cyclic imino acids (Fig. S1). The Cδ, N, Cα, and carboxyl groups of l‐proline (l‐Pro), a five‐membered imino acid, structurally resemble those of sarcosine; l‐Pro is also oxidized by Sox as a minor substrate [13, 14]. Sox derived from *Bacillus* sp. (SoxB) reacts with l‐Pro, but its *k*_cat_/*K*_m_ is approximately 1/1900‐fold that of sarcosine [7, 15]. Sox also reacts with d‐proline (d‐Pro), the optical isomer of l‐Pro. The *k*_cat_/*K*_m_ for d‐Pro is approximately 1/400‐fold that of sarcosine, and the relative efficiency is up to 4.8‐fold that of l‐Pro [7]. Thiazolidine‐4α‐carboxylic acid (thioproline, Tpr), in which the Cγ of proline is replaced by sulfur, is a minor Sox substrate with a reaction mechanism distinct from those of sarcosine and proline. Although it has higher substrate affinity than sarcosine, it exhibits substrate inhibition [16]. Although Sox reactivity toward cyclic imino acids has been examined, structural insights are lacking, hindering mechanistic understanding and enzyme engineering. To date, crystal structures of SoxB have only been reported in complexes with sarcosine [17] or various inhibitors, such as *n*‐dimethylglycine [18].

In this study, we investigated the enzymatic properties of Sox toward five‐membered ring imino acids and analyzed the structures of enzyme‐substrate complexes with d/l‐Pro and l‐Tpr. Based on the structural data obtained, we engineered mutants with enhanced reactivity toward these minor substrates and analyzed their crystal structures to elucidate the underlying reaction mechanisms. This study aimed to elucidate the structural interactions between Sox and minor substrates to improve our understanding of substrate specificity.

## Methods

### Construction of expression plasmid

A gene encoding the primary structure of SoxB (ACCESSION: D16521; Protein ID: BAA03967), with a C‐terminal His‐tag, was artificially synthesized (Life Technologies, Carlsbad, CA, USA). The nucleotide sequence was optimized according to the codon usage in *Escherichia coli*. This synthesized gene was digested with the restriction enzymes NdeI and BamHI and ligated into the pET‐29a(+) vector to generate an expression plasmid. This plasmid was used to express SoxB as a fusion protein containing a C‐terminal His‐tag. Mutant constructs were verified by DNA sequencing.

### Mutagenesis

Expression vectors for the mutants were prepared using inverse polymerase chain reaction with KOD‐plus‐ DNA polymerase (Toyobo Co., Ltd., Osaka, Japan), following the manufacturer's instructions. The DNA primers used are listed in Table S1.

### Enzyme purification

The plasmid was transformed into BL21 (DE3) competent cells (Bio Dynamic Laboratory, Tokyo, Japan) and cultured in TB medium at 37 °C with shaking at 200 rpm for 2 days. The bacterial cells were then harvested, ultrasonically disrupted, and the supernatant was collected.

The first purification was performed using a His‐tag affinity column (cOmplete His‐Tag Purification Column, Roche, Basel, Switzerland). High‐concentration fractions were selected via SDS‐PAGE (Nacalai Tesque, Kyoto, Japan), and following buffer exchange, a second purification was performed using an anion exchange column (HiTrap Q^TM^ HP Columns, GE Healthcare Sverige AB, Danderyd, Sweden). The purified eluate was buffer exchanged, concentrated, and used as the enzyme solution.

### Activity measurement

Sox enzymatic activity was quantified using a colorimetric assay based on the 4‐aminoantipyrine‐peroxidase system. Absorbance was measured using a spectrophotometer (HITACHI U‐3900; HITACHI, Tokyo, Japan). To evaluate various enzyme properties, 1 mL of working solution (50 mm Tris–HCl, pH 8.0; 0.01% 4‐aminoantipyrine; 0.02% phenol; 5 U·mL^−1^ peroxidase; X mm substrate solution) was preheated at 37 °C for 2 min, after which 35 μL of enzyme sample was added. The change in absorbance at 500 nm was monitored over 5 min.

### Calculation of kinetic parameters

The method used to calculate kinetic parameters was identical to that used for activity measurement. Initially, the *K*_m_ value was estimated, and a working solution (WS) containing five or more substrate concentrations was prepared. The substrate concentrations were arranged such that the estimated *K*_m_ value was near the center of the tested range. *K*_m_ values are typically calculated using a Lineweaver–Burk plot. However, l‐Tpr induces substrate inhibition, which can lead to an underestimation of the *K*_m_ value when using a Lineweaver–Burk plot that does not account for this effect. Therefore, in this study, the *K*_m_ and *K*_si_ values were calculated using the substrate inhibition equation [Eq. (1)] entered into Excel, with error correction performed using the Solver function. Additionally, the *k*_cat_ value was calculated by substituting the *V*_max_ value (U·mg^−1^) obtained from the Solver into Eq. (2). The same composition of WS and enzyme concentration was used to assess daily repeatability.

(1) $$V=V_{\max}\left[S\right]/\left(K_{m}+\left[S\right]+{\left[S\right]}^{2}/K_{\mathrm{si}}\right)$$

(2) $$k_{\mathrm{cat}}=V_{\max}\left(\mathrm{MW}/1000\right)\left(1/60\right)$$

In Eq. (1), [S] denotes the substrate concentration.

In Eq. (2), MW refers to the molecular weight of Sox, including the FAD cofactor (43 836 Da).

### Absorption spectrum analysis

To determine the redox state of FAD during the enzymatic reaction, a wavelength scan from 200 to 700 nm was performed using a HITACHI U‐3900 spectrophotometer (HITACHI). Solutions containing SoxB (1 mg·mL^−1^) and each substrate (8 mm, final concentration) were prepared in 50 mm Tris–HCl buffer (pH 8.0), and absorbance spectra were recorded from 0 to 180 min. The absorption spectrum of the enzyme alone (1 mg·mL^−1^) was also recorded.

### Crystallization conditions

Protein crystallization was performed using the hanging drop vapor‐diffusion method. Wild‐type SoxB crystals were obtained by mixing equal volumes (2 μL) of crystallization solution (1.35 m ammonium sulfate, 100 mm Tris–HCl buffer, pH 8.5) and protein solution (15 mg·mL^−1^ in 20 mm phosphate buffer pH 7.5) on a cover glass, followed by inversion of the sample cup. Tyr254Ala crystals were obtained using equal volumes of crystallization solution (1.6 m ammonium sulfate, 100 mm Tris–HCl buffer, pH 8.5) and protein solution (22 mg·mL^−1^ in 20 mm phosphate buffer, pH 7.5). Tyr254Gly yielded suitable crystals from equal volumes of crystallization solution (1.8 m ammonium sulfate; 100 mm Tris–HCl buffer, pH 8.5) and a protein solution (25 mg·mL^−1^; 20 mm phosphate buffer, pH 7.5). Crystals appeared after 7 days of incubation at 20 °C.

A single crystal was extracted and immersed in cryoprotectant solution (1.5 m ammonium sulfate, 100 mm Tris–HCl buffer, pH 8.5; 60 mm l‐Tpr, 477 mm l‐Pro, or 133 mm d‐Pro; 17% glycerol) for 3 min. Complex crystals were successfully obtained.

### X‐ray diffraction data and its analysis

X‐ray diffraction data were collected at the BL44XU beamline of the SPring‐8 synchrotron radiation facility (Hyogo, Japan).

Diffraction data were processed and scaled using the XDS package [19, 20], followed by further scaling with AIMLESS [21]. Initial phases were determined by molecular replacement using MOLREP from the CCP4 suite [21, 22] with the A chain of the same protein (PDB ID: 1EL5) [18] as the search model. Structural refinement was carried out using REFMAC5 from the CCP4 suite [23]. Model building was performed using Molecular Operating Environment (MOE; Ryoka Systems, Tokyo, Japan).

### Docking simulation

To investigate Sox reaction characteristics, docking simulations were performed using MOE (Ryoka Systems) with each substrate and the corresponding Sox mutant model. The crystal structures of the mutants obtained in this study served as three‐dimensional models. Sarcosine, l‐Pro, and l‐Tpr, previously studied in our laboratory, along with d‐Pro, a new substrate, were examined. Structural models of each substrate were obtained from the Protein Data Bank (PDB).

## Results

### Reactivities to sarcosine and 5‐membered ring imino acids

Five‐membered ring imino acids (l‐Pro, d‐Pro, and l‐Tpr) were selected as minor substrates for comparison with the native substrate, sarcosine. As Sox enzymes are similar to l‐amino acid oxidases [3], the l‐form is typically 50‐fold more reactive toward methyl alanine than the d‐form. However, for proline, the *V*_max_/*K*_m_ of the l‐form of SoxB was only about 1/5 of that of the d‐form [7], and the *K*_m_ of the d‐form was approximately sixfold higher (Table 1). These results indicate that the d/l selectivity of SoxB varies depending on the substrate.

**Table 1:** Kinetic parameters of wild‐type SoxB and Tyr254 mutants with each substrate. The Sox activity assay is described in the Materials and Methods section. Kinetic parameter values are expressed as mean ± standard deviation (*n* = 3). Catalytic efficiency (*k*_cat_/*K*_m_) is shown as a percentage, with the value for sarcosine (wild‐type) set at 100%. Substrate concentration was 100 mm, except for l‐thioproline (l‐Tpr) [1 mm]. n.d., not detected; n.e., not estimated; *k*_cat_ in units of s^−1^.

Substrate	Enzyme	Specific activity (U·mg^−1^)	*K*_m_ (mm)	*K*_i_ (mm)	*k*_cat_ (s^−1^)	*k*_cat_/*K*_m_ (%)
Sarcosine	Wild‐type	29 ± 3.4	17 ± 2.6	n.d.	25 ± 2.9	100
	Tyr254Ala	0.95 ± 0.18	2.4 ± 0.22	n.d.	0.54 ± 0.11	15
	Tyr254Gly	0.39 ± 0.028	34 ± 1.2	n.d.	0.40 ± 0.028	0.8
l‐Thioproline	Wild‐type	0.027 ± 0.0035	5.3 ± 0.97	4.2 ± 1.5	0.013 ± 0.0034	0.17
	Tyr254Ala	0.52 ± 0.17	0.41 ± 0.025	n.d.	0.60 ± 0.17	100
	Tyr254Gly	0.13 ± 0.020	3.1 ± 0.25	n.d.	0.37 ± 0.056	8.0
l‐Proline	Wild‐type	0.072 ± 0.090	240 ± 39	n.d.	0.18 ± 0.0233	0.05
	Tyr254Ala	0.88 ± 0.21	48 ± 2.9	n.d.	0.99 ± 0.23	1.4
	Tyr254Gly	0.64 ± 0.042	100 ± 11	n.d.	0.97 ± 0.064	0.65
d‐Proline	Wild‐type	0.13 ± 0.011	37 ± 4.2	n.d.	0.13 ± 0.011	0.24
	Tyr254Ala	0.56 ± 0.17	2.5 ± 0.27	n.d.	0.31 ± 0.0098	8.4
	Tyr254Gly	2.0 ± 0.22	6.8 ± 1.3	n.d.	1.0 ± 0.12	10
l‐Pipecolate	Wild‐type	0.67 ± 0.21	> 1600	n.d.	n.e.	n.e.
	Tyr254Ala	0.26 ± 0.019	71 ± 1.9	n.d.	0.38 ± 0.040	0.37
	Tyr254Gly	1.4 ± 0.15	16 ± 2.4	n.d.	0.74 ± 0.047	3.2
d‐Pipecolate	Wild‐type	n.d.	n.e.	n.d.	n.e.	n.e.
	Tyr254Ala	n.d.	n.e.	n.d.	n.e.	n.e.
	Tyr254Gly	0.18 ± 0.032	440 ± 47	n.d.	0.31 ± 0.015	0.00070

**Fig. 1:**
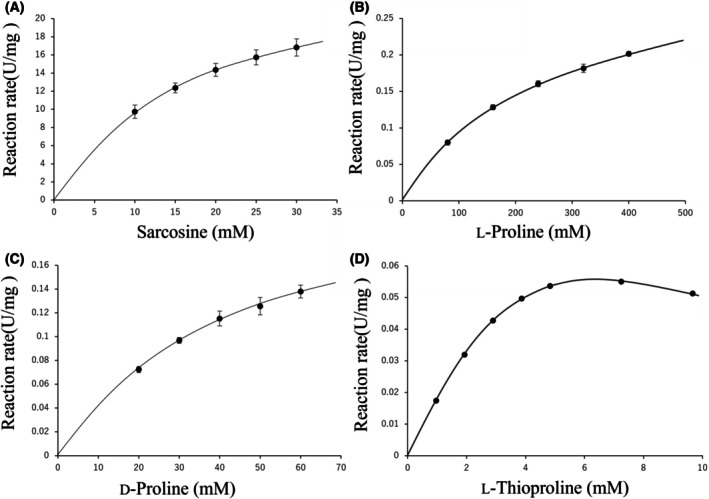
Substrate activity curves for sarcosine, l‐proline (l‐Pro), d‐proline (d‐Pro), and l‐thioproline (l‐Tpr). SoxB was used at a final concentration of approximately 0.0012 mg·mL^−1^ for sarcosine and 0.034 mg·mL^−1^ for the other substrates. The error bars represent the standard deviation of three independent experiments.

Substrate activity curves of SoxB for the four tested substrates are shown in Fig. 1. The activity curves for sarcosine, l‐Pro, and d‐Pro followed a typical Michaelis–Menten pattern. In contrast, substrate inhibition was observed only with l‐Tpr. Enzymatic activity peaked at 7 mm l‐Tpr and declined at higher concentrations (Fig. 1D).

### Spectral analysis of reaction

**Fig. 2:**
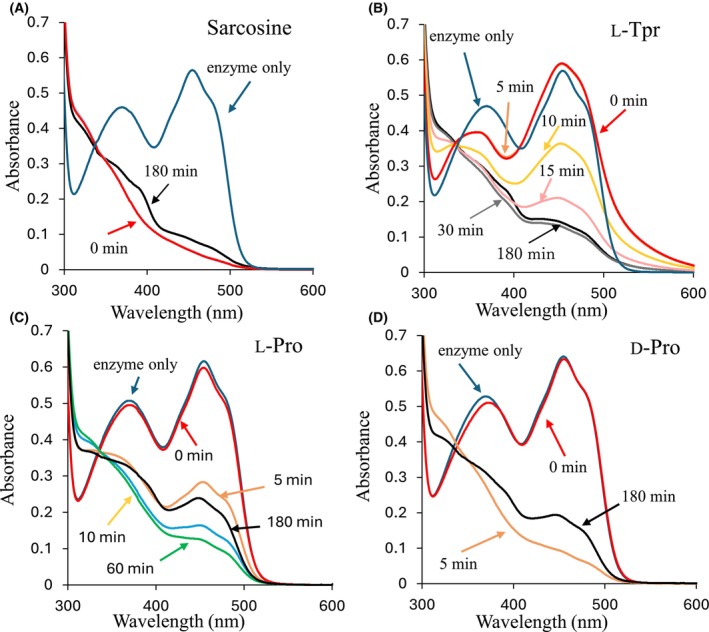
Absorption spectra for each substrate with wild‐type SoxB. Blue: enzyme only; red: 0 min after substrate addition; orange: 5 min after substrate addition; yellow: 10 min after substrate addition; black line: 180 min after substrate addition. (A) Sarcosine: no change observed from 0 to 60 min after substrate addition. (B) l‐thioproline (l‐Tpr): no change from 0 to 5 min after addition of substrate; pink: 15 min after substrate addition; gray: 30 min; no change from 30 to 60 min. (C) l‐proline (l‐Pro): green line represents 60 min. (D) d‐proline (d‐Pro): minimal change from 5 to 60 min. Each analysis used approximately 47 μmol of enzyme and 8.0 mmol of substrate. Reactions were conducted under anaerobic conditions in 50 mm Tris–HCl buffer (pH 8.0).

The Sox‐catalyzed reaction includes a reductive half‐reaction, in which the substrate is oxidized and FAD^ox^ is reduced. Subsequently, FAD^red^ is reoxidized in the oxidative half‐reaction (Fig. S2). Because the reductive half‐reaction rate of SoxB greatly exceeds the rate of oxygen diffusion into the solution, the reaction was effectively conducted under anaerobic conditions. FAD^ox^ exhibits two absorption peaks at 370 and 455 nm, which diminish upon reduction. Therefore, the reductive half‐reaction was monitored by measuring the absorption spectra of SoxB in the presence of excess substrate. Upon the addition of sarcosine, the SoxB reaction was initiated immediately, and the two FAD^ox^ peaks decreased by 49% and 91%, respectively, resulting in FAD^red^ formation (Fig. 2A). After 180 min, the peaks recovered to 60% and 15%, respectively, indicating a slow FAD^red^‐to‐FAD^ox^ conversion.

In the reaction of SoxB with l‐Tpr, a charge transfer absorption band at longer wavelengths (500 nm < *λ* < 600 nm) appeared rapidly following substrate addition (Fig. 2B). After approximately 30 min, the peaks decreased to 57% and 25%, respectively. In reactions with l‐Pro and d‐Pro, the two absorption peaks decreased within 10 and 5 min, respectively (Fig. 2B,C).

These results suggested that the reductive half‐reaction of SoxB proceeds much more slowly with the three minor substrates than with sarcosine. In particular, the reaction with l‐Tpr was extremely slow and exhibited charge transfer, a feature previously observed only in SoxB‐inhibitor interactions [18].

### Crystal structures of Sox‐substrate complexes

**Fig. 3:**
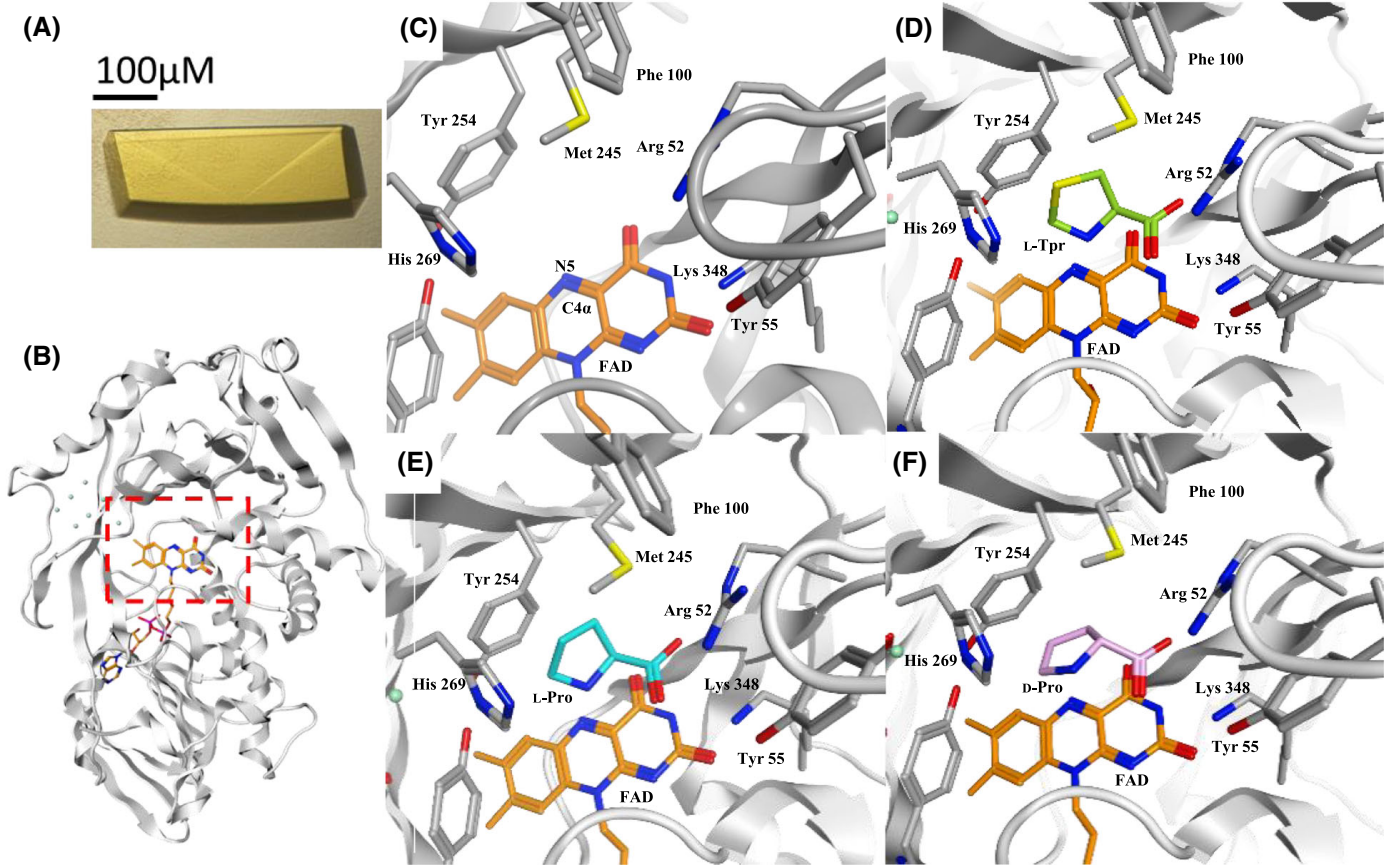
Crystal structures of SoxB and SoxB‐substrate complexes. (A) SoxB crystal. (B) SoxB open state crystal structure (Overall diagram, active site indicated by a red dashed square). (C) SoxB open state crystal structure [close‐up of active site]. (D) Complex crystal structure of l‐thioproline (l‐Tpr) [close‐up of active site]. (E) Complex crystal structure of l‐proline (l‐Pro) [close‐up of active site]. (F) Complex crystal structure of d‐proline (d‐Pro) [close‐up of active site]. Atoms are colored as follows: oxygen, red; nitrogen, blue; sulfur, yellow. Carbon and water colors vary by context. The active site is composed of seven amino acid residues (Arg52, Tyr55, Phe100, Met245, Tyr254, His269, and Lys348) surrounding the isoalloxazine ring of the FAD coenzyme.

To elucidate the mechanism of substrate inhibition by l‐Tpr, X‐ray crystallographic analysis was performed. The yellow crystals of ligand‐free SoxB indicated the presence of FAD^ox^ (Fig. 3A). Crystals of SoxB complexed with l‐Tpr (SoxB–l‐Tpr complex, PDB ID: 9U3B), l‐Pro (SoxB–l‐Pro complex, PDB ID: 9U3C), or d‐Pro (SoxB–d‐Pro complex, PDB ID: 9U3D) belonged to space group P2_1_. Diffraction data were collected at resolutions of 1.30, 1.54, and 1.35 Å, respectively (Table S2). Initial phases were determined using molecular replacement with the SoxB‐dimethylglycine complex (PDB ID: 1EL5) [18] as the search model.

X‐ray crystallography data showed that each substrate was anchored near the N5 and C4x atoms of FAD by Arg52, Tyr55, and Lys348 (Fig. 3D–F).

**Fig. 4:**
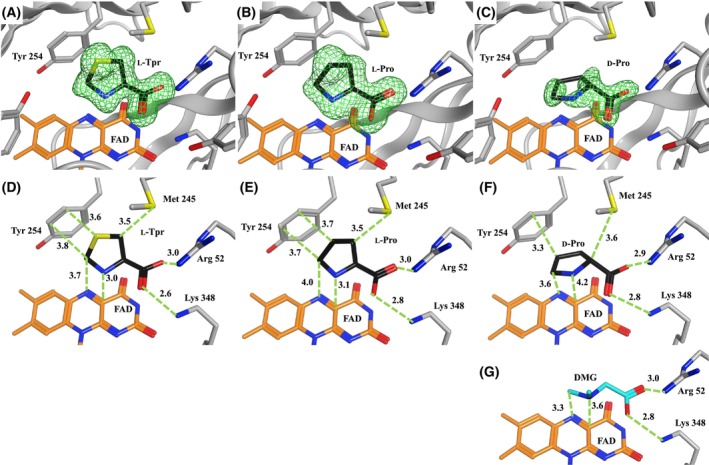
Distance between active site residues and substrates. (A, D) SoxB l‐thioproline (l‐Tpr) complex. (B, E) SoxB l‐proline (l‐Pro) complex. (C, F) SoxB d‐proline (d‐Pro) complex. Black stick: bound substrate. (G) SoxB‐DMG complex (light blue stick: DMG; PDB ID: 1EL5). The *F*_o_ *–F*_c_ omit map is contoured at 3σ around the bound substrate. Oxygen atoms are shown in red; nitrogen in blue; sulfur in yellow; and FAD carbon in orange. Numbers indicate interatomic distance in Å. DMG: *N*‐dimethylglycine.

The *F*_o_–*F*_c_ omit maps (Fig. 4A–C) confirmed that l‐Tpr, l‐Pro, and d‐Pro were located in the active site. The Cδ of l‐Tpr is 3.7 Å from the N5 of FAD, and the nitrogen atom is 3.0 Å from the C4x atom, which are predicted to be involved in the reaction [24]. l‐Tpr is constrained by Arg52 and Lys348, and Tyr254 at distances of 3.0 and 2.6 Å, respectively, and lies 3.6 Å from Tyr254 (Fig. 4D). The C‐Cα‐Cβ‐S torsion angle of bound l‐Tpr is −104°, and the C‐Cα‐N‐Cδ torsion angle is 133°. The Cδ of l‐Pro is 4.0 Å from the N5 of FAD, and the nitrogen is 3.1 Å from the C4x atom, both of which are involved in the catalytic reaction. l‐Pro is constrained by Arg52 and Lys348 at distances of 3.0 and 2.8 Å, respectively, and lies 3.7 Å from the nearest atom of Tyr254 (Fig. 4E). The C‐Cα‐Cβ‐Cγ torsion angle of the bound l‐Pro is −102°, and the C‐Cα‐N‐Cδ angle is 127°. The Cδ of d‐Pro is 3.6 Å from the N5 atom of FAD, and the nitrogen is 4.2 Å from the C4x atom, both involved in the predicted direct reaction. d‐Pro is constrained by Arg52 and Lys348 at distances of 2.9 and 2.8 Å, respectively, and lies 3.3 Å from Tyr254 (Fig. 4F). The C‐Cα‐Cβ‐Cγ torsion angle of the bound d‐Pro is 134°, and the C‐Cα‐N‐Cδ angle is −127°. These substrates were positioned in close proximity to Tyr254 and were oriented for potential catalytic reaction. Additionally, *n*‐dimethylglycine (DMG), a sarcosine analog known to bind at the same position [18], has been previously analyzed. It is constrained by Arg52 and Lys348 at distances of 3.0 and 2.8 Å, respectively, and is positioned 3.3 Å from the C5 and N5 atoms of FAD, and 3.6 Å from the nitrogen and C4x atoms of FAD.

### Kinetic properties of Tyr254 mutants

As shown in Fig. 4D,E, the five‐membered rings of l‐Pro and l‐Tpr were approximately 3.7 Å from the side chain of Tyr254. At this distance, van der Waals repulsive interactions may occur. Therefore, SoxB mutants with substitutions at residue Tyr254 were constructed. Among these, Tyr254Ala and Tyr254Gly were selected for their enhanced reactivity toward l‐Tpr.

The Tyr254 mutant enzymes were purified, and their kinetic parameters of sarcosine, l‐Pro, d‐Pro, and l‐Tpr were determined and compared with those of the wild‐type enzyme (Table 1). The reactivities toward l‐pipecolate and d‐pipecolate, minor substrates with larger ring sizes, were also examined. When cyclic imino acids were used as substrates, significant increases in specific activity were observed for the Tyr254Ala and Tyr254Gly mutants. In particular, the specific activities of the Tyr254Ala mutant were approximately 12‐ and 19‐fold higher for l‐Pro and l‐Tpr respectively, compared with those of the wild‐type. Similarly, the Tyr254Gly mutant exhibited an approximately 15‐fold increase in specific activity for d‐Pro compared with the wild‐type. The specific activity for sarcosine was higher in the wild‐type enzyme than in the SoxB mutants. Catalytic efficiencies toward cyclic imino acids were higher in the SoxB mutants than in the wild‐type, whereas the catalytic efficiency toward sarcosine was greater in the wild‐type enzyme.

### Spectral analysis of Tyr254 mutants

**Fig. 5:**
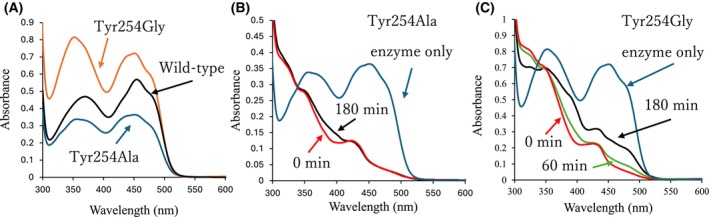
Absorption spectra of SoxB and its mutants with l‐thioproline (l‐Tpr). (A) Comparison of absorption spectra among SoxB wild‐type (black), Tyr254Ala (blue), and Tyr254Gly (orange). (B) Absorption spectrum of Tyr254Ala with L‐Tpr. Blue: enzyme only; red: 0 min after substrate addition; no change from 0 to 60 min; black: 180 min. (C) Absorption spectrum of Tyr254Gly with L‐Tpr. Blue: enzyme only; red: 0 min; green: 60 min; black: 180 min. Approximately 47 μmol of enzyme and 8.0 mmol of l‐Tpr were used in each assay. All reactions were conducted under anaerobic conditions in 50 mm Tris–HCl buffer (pH 8.0).

The absorption spectra of the mutant SoxB proteins differed from those of the wild‐type. The wild‐type spectrum showed peaks at approximately 370 and 455 nm, characteristic of oxidized FAD. However, the Tyr254Ala and Tyr254Gly mutants exhibited altered absorption peaks (Fig. 5A). Tyr254Ala exhibited peaks at 356 and 450 nm, with reduced absorbance relative to the wild‐type. In contrast, the Tyr254Gly peaks were slightly shifted (352 and 451 nm) and showed increased absorbance compared to the wild‐type (Fig. 5A).

The absorption spectra of l‐Tpr in the Tyr254Ala and Tyr254Gly mutants were measured (Fig. 5B,C). Immediately after l‐Tpr addition, the two peaks in both mutants nearly disappeared, indicating rapid reduction in FAD^ox^. Peak recovery was observed at 180 and 60 min for Tyr254Ala and Tyr254Gly, respectively, indicating conversion of FAD^red^ to FAD^ox^. Unlike the wild‐type (Fig. 2A), both mutants lacked initial charge transfer absorption at longer wavelengths, and the peaks disappeared immediately.

### Crystal structures of Tyr254 mutants

X‐ray crystallography was performed for the Tyr254Ala and Tyr254Gly mutants. Yellow crystals, similar to those of the wild‐type (Fig. 3A), indicated that FAD was in the oxidized form. Diffraction data were obtained at resolutions of 1.37–1.45 Å, with both structures belonging to space group *P*2_1_ (Table S3). Initial phases were determined by molecular replacement using the SoxB‐l‐Tpr complex as the model.

**Fig. 6:**
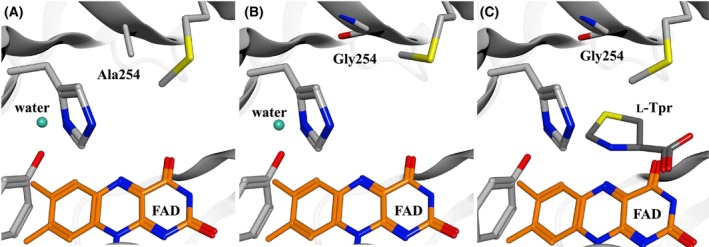
Active site structures of SoxB mutants Tyr254Ala and Tyr254Gly. (A) Active site of Tyr254Ala. (B) Active site of Tyr254Gly. (C) Docking simulation of the Tyr254Gly‐l‐Tpr complex.

Complexes of the mutants with substrates, including l‐Tpr, were generated and analyzed. However, structural analysis revealed the absence of l‐Trp at the active site, likely due to the elimination of substrate inhibition and increased reactivity. Therefore, docking simulations were performed using MOE. The Tyr254Ala and Tyr254Gly mutants showed minimal differences in the positions of active site side chains compared with the wild‐type. In both mutants, the active site cavity was significantly expanded (Fig. 6A,B). Docking models showed that in the mutants, l‐Tpr could enter the active site without steric interference (Fig. 6C).

**Fig. 7:**
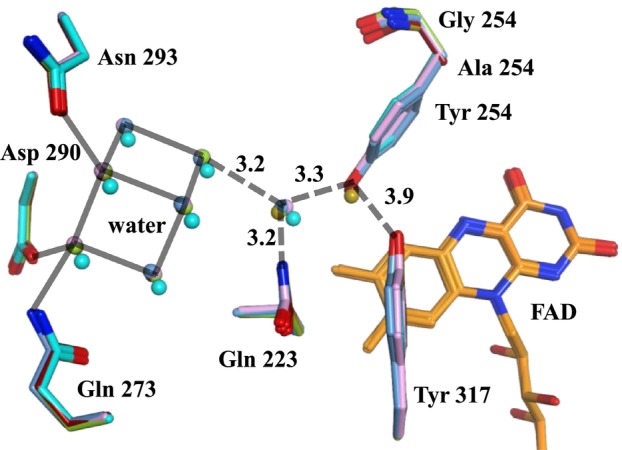
Comparison of the water network between wild‐type and mutant SoxB enzymes. Gray: SoxB wild‐type; blue: SoxB wild‐type [substrate: DMG]; green: SoxB wild‐type [substrate: l‐thioproline (l‐Tpr)]; light blue: SoxB wild‐type [substrate: l‐proline (l‐Pro)]; pink: SoxB wild‐type [substrate: d‐proline (d‐Pro)]; dark red: SoxB Tyr254Ala; earth color: Tyr254Gly. Spheres indicate water molecules. Solid lines represent hydrogen bonds at distances of ~2.8 Å. Dotted lines indicate hydrogen bonds with corresponding distances labeled (units = Å).

In both the SoxB structures determined in this study and the model (PDB ID: 1EL5), a water network of seven molecules was located at nearly identical positions (Fig. 7). In the 1EL5 structure, network water molecules exhibited lower B‐factors (approximately 16 Å^2^) compared with bulk water molecules (approximately 34 Å^2^). This pattern persisted in 9U3B, where network water molecules had B‐factors of approximately 13 Å^2^ versus 25 Å^2^ for bulk water molecules. This trend continued in 9U3C, 9U3D, 9U3E, and 9U3F, where network water consistently exhibited B‐factors about half those of the corresponding bulk water molecules. In the Tyr254Ala and TyrR254Gly mutants, a water molecule replaced the hydroxyl oxygen atom of Tyr254 observed in the wild‐type protein.

## Discussion

### Structured properties around the active site

Sox recognizes substances with sarcosine‐like structures and cleaves the bonds between the nitrogen atom and the carbon atom on the methyl group. Although ethylglycine and its methylated amino acids are suitable substrates, their affinity and reactivity are lower than those of sarcosine [5]. The bulky functional groups are expected to interfere with reaching the optimal position for the reaction.

Sox family enzymes conserve three residues: Arg52, Tyr55, and Lys348, with residue numbers corresponding to SoxB. These residues form salt bridges with the substrate's carboxyl group, stabilizing position [3]. Additionally, three residues, Phe100, Met245, and Tyr254, form the substrate pocket. *N*‐Methyltryptophan oxidase (MTox), an enzyme in the same family as Sox, primarily uses methyltryptophan as a substrate but also reacts with sarcosine. When Thr239 of MTox, corresponding to Met245 of Sox, was substituted with Met, activity toward sarcosine increased, while activity toward methyltryptophan decreased [25]. In Sox from *Arthrobacter* sp. (SoxA), replacing Phe103 with an amino acid possessing a smaller side chain reduced activity toward sarcosine [26]. Molecular modeling suggested that Phe103 does not directly affect the active site but influences activity by limiting the movement of Met248 [27]. Since SoxB also contains Phe100 and Met245 at the same positions as SoxA, Phe100 in SoxB is speculated to be homologous to Phe103 of SoxA. His269 also contributes to maintaining a hydrophilic environment and spatial configuration at the active site [28]. The active site of SoxB is thought to be primarily influenced by these seven residues (Fig. 3C).

### d/l substrate selectivity

This study provides new insights into the structure of Sox–substrate complexes. Wild‐type Sox generally favors the l‐form in d/l selection [3]; however, this preference is substrate‐dependent (Table 1). The d‐form is more reactive than the l‐form for proline, while only the l‐form reacts with pipecolate. The key difference between these substrates is that proline is a five‐membered ring imino acid, whereas pipecolate is a six‐membered ring imino acid. The d‐form of a five‐membered ring can bind in a relatively planar conformation. Conversely, the l‐form cannot access the active site unless its conformation is adjusted by Tyr254 (Fig. 4A–C). Based on these findings, the reactivity of six‐membered cyclic imino acids was inferred. However, docking simulations revealed that the six‐membered cyclic imino acids bind in a non‐traditional manner: d‐pipecolate adopts a high‐energy half‐chair conformation, whereas l‐pipecolate assumes a chair conformation. Therefore, d‐pipecolate is presumed to exhibit unstable binding and rapidly dissociates from the enzyme active site (data not shown).

### Substrate structures in the complexes

The Sox‐bound and free substrates were compared by superimposing the α carbon, the carbon atoms of the carboxy group, and the oxygen atom using MOE software (Fig. S3A). The conformation of the five‐membered ring in Sox‐bound d‐Pro was nearly identical to that in free d‐Pro, whereas l‐Tpr and l‐Pro showed significant conformational differences between the bound and free states. The dihedral angles of the five‐membered ring of l‐Tpr changed by 42° and 34°, and those of l‐Pro changed by 42° and 33°, respectively. The Sox‐bound conformations of l‐Tpr and l‐Pro appeared to be unstable. These altered five‐membered rings were positioned near the tip of Tyr254 and partially aligned parallel to its phenolic ring (Fig. S3B). Therefore, the presence of Tyr254 allows the five‐membered rings of l‐Tpr and l‐Pro to enter the active site in this bent conformation. This increases the distance between the Cδ of the substrate and the N5 atom of FAD to approximately 4 Å, which may explain why these substrates exhibit low reactivity as minor substrates.

### Minor substrate reactions

Due to differing distances between the substrates and FAD, the reaction mechanisms may also vary. For example, the SoxB reaction of SoxB with sarcosine has been proposed to follow the HACET mechanism. When the DMG–SoxB complex (PDB ID: 1EL5) [18] was considered as a model for the sarcosine–SoxB complex, d‐Pro showed a similar binding mode (Fig. 4F,G). However, the distances between the predicted reactive atoms of FAD and d‐Pro were increased by 0.3 Å and 0.6 Å, respectively. These differences may alter the electron transfer rate, and the reaction may proceed via a carbanion mechanism [29], in which the substrate Cδ binds to N5 of FAD followed by the electron transfer, rather than the HACET mechanism, where electrons are simultaneously transferred from the substrate Cδ and nitrogen to N5 and C4x of FAD. Unlike sarcosine, l‐Pro and l‐Tpr have their nitrogen atoms positioned closer to FAD than to their respective Cδ. These two substrates may also react via the HACET mechanism. However, since the distance between Cδ and N5 differs by approximately 1 Å from that between the nitrogen and C4x, it is possible that the nitrogen first binds to C4x of FAD, followed by electron transfer, as in a polar mechanism [10, 11]. Our previous studies demonstrated that SoxB's reaction with *n*‐cyclopropylglycine proceeds via a polar mechanism rather than the HACET mechanism, as shown by fragment molecular orbital, quantum mechanical, and molecular mechanical calculations [12]. Further studies using these approaches are needed to validate these findings.

### Mutational effects on the enzyme reaction

The Tyr254Ala and Tyr254Gly mutants exhibited significantly increased enzymatic activity toward cyclic imino acids compared with the wild‐type and were able to react with d‐pipecolate, unlike the wild‐type. We confirmed that the side chain of Tyr254 causes spatial interference with the cyclic imino acid substrate, thereby inhibiting the enzymatic reaction. Although substrate affinity did not change significantly, enzymatic reactivity toward sarcosine, the native substrate, was lower than that of the wild‐type (Table 1). Tyr254 plays a role in forming the active site. Therefore, when its bulky phenolic ring is removed (Fig. 6A,B), sarcosine can no longer be stabilized in a favorable position for the reaction, resulting in decreased reactivity.

The Tyr254Ala and Tyr254Gly mutants were no longer inhibited by l‐Tpr, and their reactivity was significantly improved. Charge transfer at long wavelengths, characteristic of l‐Tpr substrate inhibition, was not observed in the absorption spectra of the mutants. The presence of Tyr254 is thought to be responsible for the substrate inhibition of SoxB by l‐Tpr. However, although l‐Pro is also spatially interfered with by Tyr254, no substrate inhibition was observed (Fig. S3B). Therefore, substrate inhibition of l‐Tpr likely involves factors beyond interference from Tyr254. The only structural difference between l‐Tpr and l‐Pro is the presence of a sulfur atom in l‐Pro. The distance between the sulfur atom of l‐Tpr and that of Met245 is 3.86 Å, and the distance to the Cε of Met245 is 3.74 Å. This indicates that interactions can occur between the sulfur of l‐Tpr and the side chain of Met245. Therefore, electron delocalization is thought to occur between the Met245 side chain and the sulfur, Cδ, and nitrogen atoms of l‐Tpr and the C4x atom of FAD [30]. This promotes charge transfer, which hinders the reaction and causes substrate inhibition. In the mutants, the sulfur atom of l‐Tpr no longer remains close to Met245; thus, electron delocalization is suppressed, consistent with the experimental results showing that charge transfer cannot occur (Table 1).

In the Tyr254Ala and Tyr254Gly mutants, the hydroxyl oxygen atom of Tyr254 in the wild‐type is replaced by a water molecule, suggesting that the water network contributes to stabilizing the Tyr254 side chain.

### Practical utility

Sox is already used in clinical testing, particularly for measuring creatinine clearance, a renal function marker [8, 31]. Increased blood creatinine levels indicate a decline in kidney function [32]. In enzyme assays, Sox's reactivity toward proline can lead to false‐positive results. This issue has been addressed by developing enzymes with improved substrate specificity through random and site‐directed mutagenesis [15]. This study utilized structural insights to explain the d/l selectivity of Sox and demonstrated that enlarging the active site cavity reduces substrate inhibition. These findings may aid in designing future Sox mutants and refining diagnostic agents.

## Conclusion

In this study, we constructed substrate activity curves for SoxB and l‐Tpr and measured the absorption spectra of l‐Tpr, l‐Pro, and d‐Pro. The enzyme‐substrate complexes of SoxB with l‐Tpr, l‐Pro, and d‐Pro were crystallized, and their structures were analyzed. To our knowledge, this is the first report to determine the tertiary structure of oxidized Sox‐substrate complexes. We hypothesized that Tyr254 in the reaction center affects reactivity with five‐membered cyclic imino acids and constructed the Tyr254Ala and Tyr254Gly mutants. The mutants exhibited higher reactivity toward five‐membered cyclic imino acids than the wild‐type. Structural analysis revealed that the active site in the mutants was enlarged, facilitating the reaction with five‐membered cyclic imino acids. These findings will be useful for understanding the causes of false positives in enzyme assays and in the development of enzymes with high substrate specificity. In this study, the reaction mechanisms between Sox and minor substrates were not fully elucidated; therefore, further experiments using computational approaches are currently underway.

## Conflicts of interest

The authors declare no conflicts of interest.

## Peer review

The peer review history for this article is available at https://www.webofscience.com/api/gateway/wos/peer‐review/10.1002/2211‐5463.70119.

## Author contributions

YZ and YNi conceived and designed the project; TH and YNi supervised the study; YZ and MK performed the experiments; YZ, YNa, MK, TH, and YNi analyzed and interpreted the data; YZ, YNa, TH, and YNi wrote the manuscript; YZ, YNa, MK, TN, TH, and YNi discussed the results and contributed to the final manuscript.

## Supporting information


**Table S1.** Primer sequence used for inverse PCR‐based mutant construction.
**Table S2.** X‐ray diffraction data collection and refinement statistics for SoxB‐substrate complexes.
**Table S3.** X‐ray diffraction data collection and refinement statistics for SoxB mutants.
**Fig. S1.** Sox substrates. Sarcosine (PubChem CID: 1088), l‐proline (PubChem CID: 145742), d‐proline (PubChem CID: 8988), l‐thioproline (PubChem CID: 93176), l‐pipecolic acid (PubChem CID: 439227), d‐pipecolic acid (PubChem CID: 736316).
**Fig. S2.** Schematic of the Sox reaction.
**Fig. S3.** Effect of Tyr254 on substrate blinding. (A) Superposition of Sox‐bound and free substrate structures. (B) Parallel orientation of five‐membered ring imino acids relative to the six‐membered ring of Tyr254. The black stick indicates the bound substrate; the gray stick indicates the free substrate. Oxygen is red, nitrogen is blue, and sulfur is yellow. Orange: parallel planes.

## Data Availability

The structural data obtained in this study have been deposited in the Protein Data Bank under accession codes 9U3B, 9U3C, 9U3D, 9U3E, and 9U3F.

## References

[1] Ikushiro H (2015) Mechanistic enzymology of serine palmitoyltransferase–stereochemical reaction control revealed by the side reaction of mutant enzymes. J Biochem 87, 298–307. 26571594

[2] Obata D , Takabayashi A , Tanaka R , Tanaka A and Ito H (2019) Horizontal transfer of promiscuous activity from nonphotosynthetic bacteria contributed to evolution of chlorophyll degradation pathway. Mol Biol Evol 36, 2830–2841. 31432082 10.1093/molbev/msz193

[3] Lahham M , Jha S , Goj D , Macheroux P and Wallner S (2021) The family of sarcosine oxidases: same reaction, different products. Arch Biochem Biophys 704, 108868. 33812916 10.1016/j.abb.2021.108868

[4] Zeller HD , Hille R and Jorns MS (1989) Bacterial sarcosine oxidase: identification of novel substrates and a biradical reaction intermediate. Biochemistry 28, 5145–5154. 2475174 10.1021/bi00438a035

[5] Wagner MA and Jorns MS (2000) Monomeric sarcosine oxidase: 2. Kinetic studies with sarcosine, alternate substrates, and a substrate analogue. Biochemistry 39, 8825–8829. 10913293 10.1021/bi000350y

[6] Dodt G , Kim DG , Reimann SA , Reuber BE , McCabe K , Gould SJ and Mihalik SJ (2000) L‐pipecolic acid oxidase, a human enzyme essential for the degradation of L‐pipecolic acid, is most similar to the monomeric sarcosine oxidases. Biochem J 345, 487–494. 10642506 PMC1220782

[7] Nishiya Y , Nakano S , Kawamura K and Abe Y (2012) Monomeric sarcosine oxidase acts on both L‐ and D‐substrates. Int J Anal Bio‐Sci 35, 426–430.

[8] Nishiya Y and Imanaka T (1993) Cloning and sequencing of the sarcosine oxidase gene from *Arthrobacter* sp. TE1826. J Ferment Bioeng 75, 239–244.

[9] Abe Y , Shoji M , Nishiya Y , Aiba H , Kishimoto T and Kitaura K (2017) The reaction mechanism of sarcosine oxidase elucidated using FMO and QM/MM methods. Phys Chem Chem Phys 19, 9811–9822. 28374027 10.1039/c6cp08172j

[10] Kim JM , Bogdan MA and Mariano PS (1993) Mechanistic analysis of the 3‐methyllumiflavin‐promoted oxidative deamination of benzylamine. A potential model for monoamine oxidase catalysis. J Am Chem Soc 115, 10591.

[11] Kim JM , Hoegy SE and Mariano PS (1995) Flavin chemical models for monoamine oxidase inactivation by cyclopropylamines, alpha.‐Silylamines, and hydrazines. J Am Chem Soc 117, 100–105.

[12] Shoji M , Abe Y , Boero M , Shigeta Y and Nishiya Y (2020) Reaction mechanism of * n *‐cyclopropylglycine oxidation by monomeric sarcosine oxidase. Phys Chem Chem Phys 22, 561. 10.1039/d0cp01679a 32452478

[13] Nishiya Y (2016) Similarities and individualities of flavin adenine dinucleotidelinked oxidases for diagnostic use. Int J Anal Bio‐Sci 39, 189–194.

[14] Ohsawa S , Mutoh T , Mamada K , Yoshida T , Iida S and Yonemitsu H (1994) Evaluation of an enzymatic reagent for the determination of creatinine. Int J Anal Bio‐Sci 17, 332–337.

[15] Nishiya Y and Kishimoto T (2010) Alteration of L‐proline oxidase activity of sarcosine oxidase and a structural interpretation. Int J Anal Bio‐Sci 33, 161–166.

[16] Nishiya Y , Yoshida C and Okajima T (2016) A new substrate for monomeric sarcosine oxidase selected by *in silico* analysis. Int J Anal Bio‐Sci 4, 83–91.

[17] Kommoju PR , Chen Z , Bruckner RC , Mathews FS and Jorns MS (2011) Probing oxygen activation sites in two flavoprotein oxidases using chloride as an oxygen surrogate. Biochemistry 50, 5521–5534. 21568312 10.1021/bi200388g PMC3448946

[18] Wagner MA , Trickey P , Chen Z , Scott MF and Jorns MS (2000) Monomeric sarcosine oxidase: 1. Flavin reactivity and active site binding determinants. Biochemistry 39, 8813–8824. 10913292 10.1021/bi000349z

[19] Kabsch W (2010b) XDS. Acta Crystallogr D Biol Crystallogr 66, 125–132. 20124692 10.1107/S0907444909047337 PMC2815665

[20] Kabsch W (2010a) Integration, scaling, space‐group assignment and post‐refinement. Acta Crystallogr D Biol Crystallogr 66, 133–144. 20124693 10.1107/S0907444909047374 PMC2815666

[21] Winn MD , Ballard CC , Cowtan KD , Dodson EJ , Emsley P , Evans PR , Keegan RM , Krissinel EB , Andrew L , McCoy A *et al*. (2011) Overview of theCCP4 suite and current developments. Acta Crystallogr D Biol Crystallogr 67, 235–242. 21460441 10.1107/S0907444910045749 PMC3069738

[22] Vagin A and Teplyakov A (2009) Molecular replacement with MOLREP. Acta Crystallogr D Biol Crystallogr 66, 22–25. 20057045 10.1107/S0907444909042589

[23] Murshudov GN , Skubák P , Lebedev AA , Pannu NS , Steiner RA , Nicholls RA , Winn MD , Long F and Vagin AA (2011) REFMAC5 for the refinement of macromolecular crystal structures. Acta Crystallogr D Biol Crystallogr 67, 355–367. 21460454 10.1107/S0907444911001314 PMC3069751

[24] Trickey P , Wagner MA , Jorns MS and Mathews FS (1999) Monomeric sarcosine oxidase: structure of a covalently flavinylated amine oxidizing enzyme. Structure 7, 331–345. 10368302 10.1016/s0969-2126(99)80043-4

[25] Ilari A , Bonamore A , Franceschini S , Fiorillo A , Boffi A and Colotti G (2008) The X‐ray structure of *n*‐methyltryptophan oxidase reveals the structural determinants of substrate specificity. Proteins 71, 2065–2075. 18186483 10.1002/prot.21898

[26] Nishiya Y and Imanaka T (1994) Alteration of substrate specificity and optimum pH of sarcosine oxidase by random and site‐directed mutagenesis. Appl Environ Microbiol 60, 4213–4215. 16349451 10.1128/aem.60.11.4213-4215.1994 PMC201966

[27] Nishiya Y (2013) Altered substrate affinity of monomeric sarcosine oxidase by mutation of phenylalanine‐103 or histidine‐348. Int J Anal Bio‐Sci 1, 21–26.

[28] Zhao G , Song H , Chen Z , Scott MF and Jorns MS (2002) Monomeric sarcosine oxidase: role of histidine 269 in catalysis. Biochemistry 41, 9751–9764. 12146941 10.1021/bi020286f

[29] Harris RJ , Meskys R , Sutcliffe MJ and Scrutton NS (2000) Kinetic studies of the mechanism of carbon−hydrogen bond breakage by the heterotetrameric sarcosine oxidase of *Arthrobacter* sp. 1‐IN. Biochemistry 39, 1189–1198. 10684595 10.1021/bi991941v

[30] Batsanov SS (2001) Van der Waals Radii of elements. Inorg Mater 37, 871–885.

[31] Nishiya Y , Zuihara S and Imanaka T (1995) Active site analysis and stabilization of sarcosine oxidase by the substitution of cysteine residues. Appl Environ Microbiol 61, 367–370. 7887617 10.1128/aem.61.1.367-370.1995 PMC167291

[32] Horio M (2007) Evaluation of kidney function. Nippon Naika Gakkai Zasshi 96, 159–165. 17305071 10.2169/naika.96.159

