# Supplementary material for: Structural and functional analysis of Bacillus sarcosine oxidase and its activity toward cyclic imino acids
Source: FEBS Open Bio. 2025 Sep 11;15(11):1814–26. doi: 10.1002/2211-5463.70119 (PMC12582975; doi:10.1002/2211-5463.70119)
Supplement: Supplementary file 1 — Table S1. Primer sequence used for inverse PCR‐based mutant construction. Table S2. X‐ray diffraction data collection and refinement statistics for SoxB‐substrate complexes. Table S3. X‐ray diffraction data collection and refinement statistics for SoxB mutants. Fig. S1. Sox substrates. Sarcosine (PubChem CID: 1088), l‐proline (PubChem CID: 145742), d‐proline (PubChem CID: 8988), l‐thioproline (PubChem CID: 93176), l‐pipecolic acid (PubChem CID: 439227), d‐pipecolic acid (PubChem CID: 736316). Fig. S2. Schematic of the Sox reaction. Fig. S3. Effect of Tyr254 on substrate blinding. (A) Superposition of Sox‐bound and free substrate structures. (B) Parallel orientation of five‐membered ring imino acids relative to the six‐membered ring of Tyr254. The black stick indicates the bound substrate; the gray stick indicates the free substrate. Oxygen is red, nitrogen is blue, and sulfur is yellow. Orange: parallel planes. [file FEB4-15-1814-s001.docx]

**Supporting Information**

**Table S1.** Primer sequence used for inverse PCR-based mutant construction.**
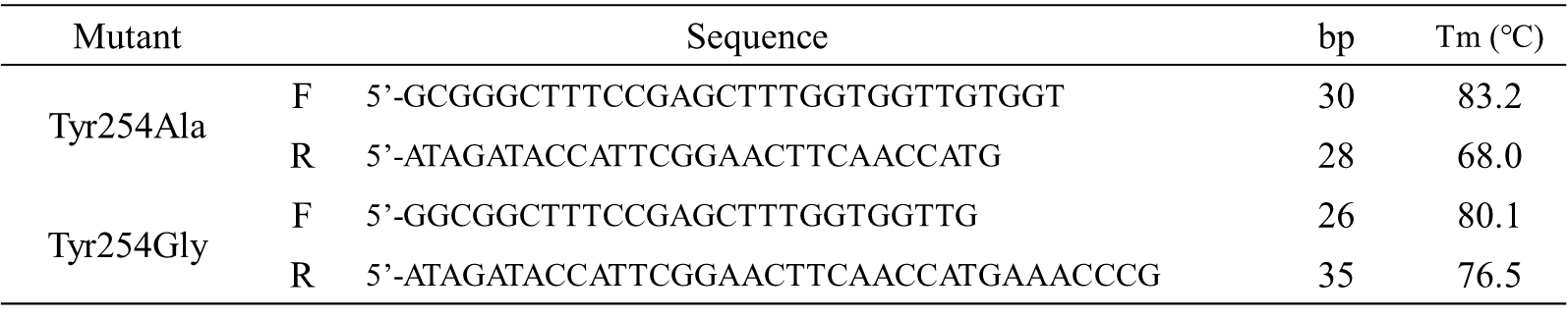
**

F and R indicate forward and reverse primers, respectively. Mutation sites are underlined.

**
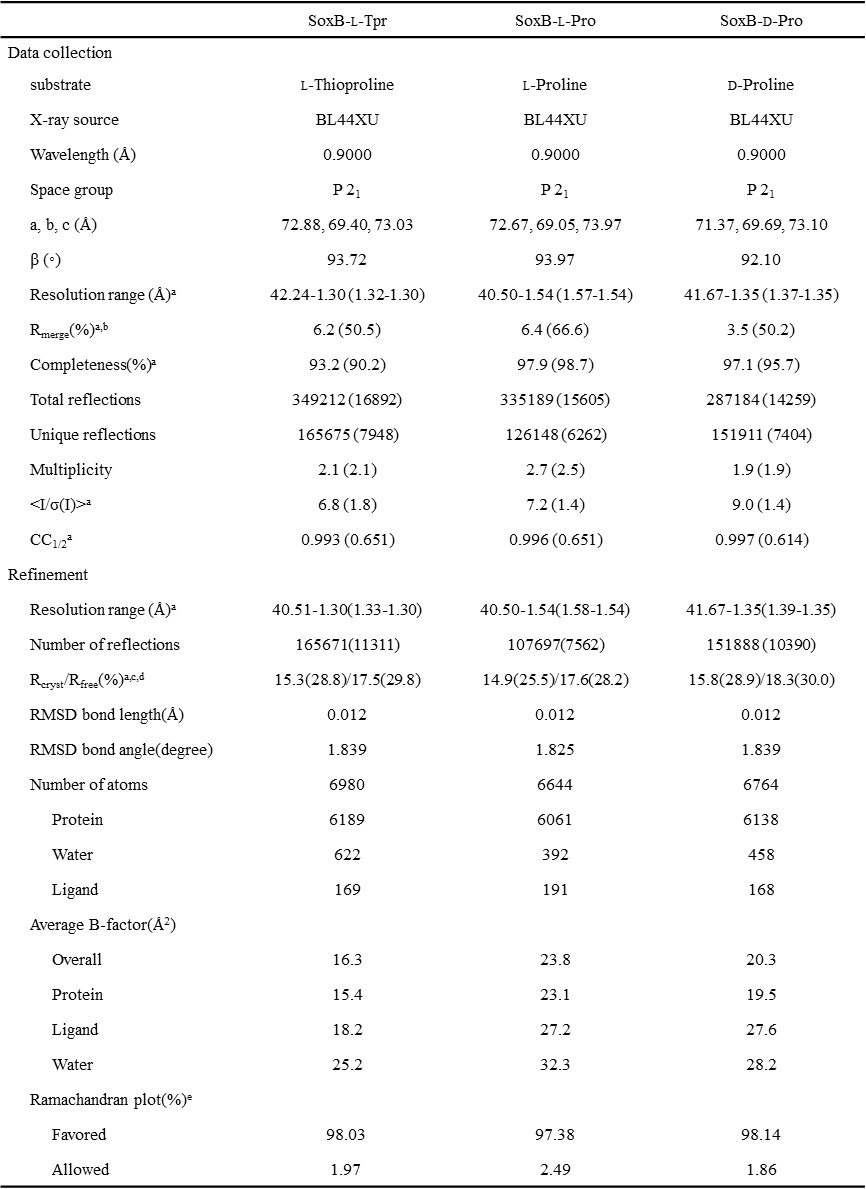
 Table S2**. X-ray diffraction data collection and refinement statistics for SoxB-substrate complexes.

^a^ Values in parentheses are for the highest resolution shell.

^b^*R*_merge_ = Σ_hkl_ Σ_i_ |*I*_hkl,j_ - <*I*_hkl_>| / Σ_hkl_ Σ_i_ *I*_hkl,j_, where *I*_hkl,j_ is the intensity of observation *I*_hkl,j_ and <*I*_hkl_> is the average of symmetry-related observations of a unique reflection.

^c^*R*_cryst_ = Σ||*F*_o_| - |*F*_c_|| / Σ|*F*_o_|, where *F*_o_ and *F*_c_ are observed and calculated structure factor amplitudes, respectively.

^d^*R*_free_ was calculated using a randomly selected 5% of the dataset that was omitted from all stages of refinement.

^e^Ramachandran plots were prepared for all residues other than Gly and Pro.

**
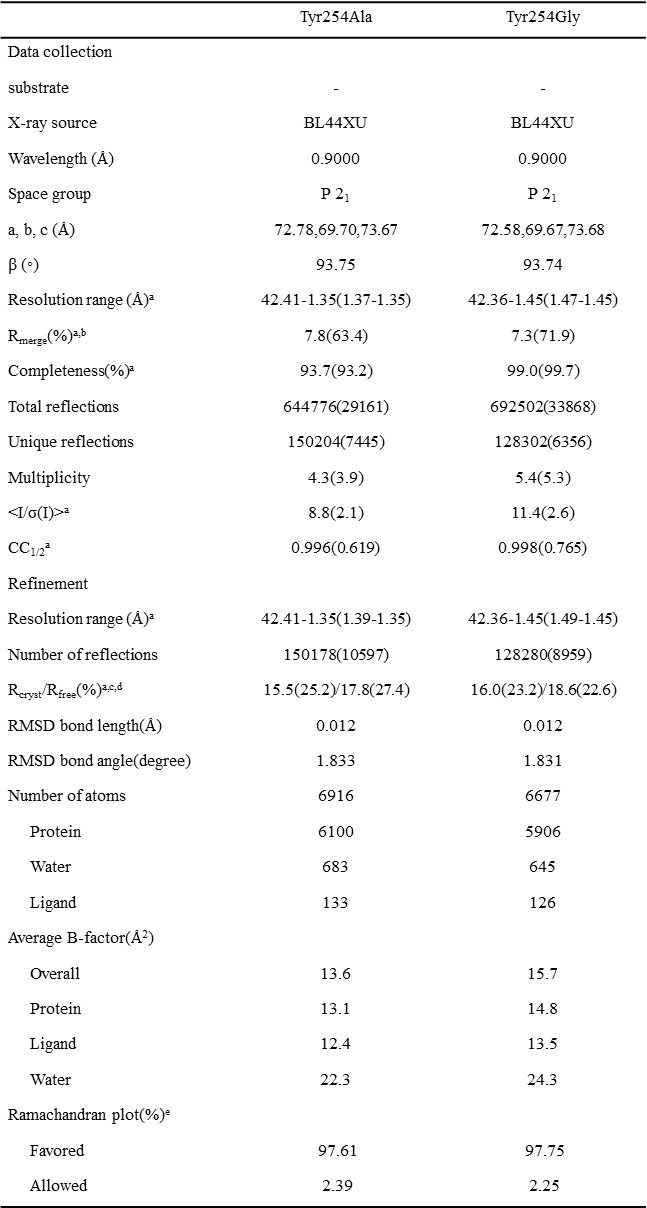
Table S3**. X-ray diffraction data collection and refinement statistics for SoxB mutants.

^a^ Values in parentheses are for the highest resolution shell.

^b^*R*_merge_ = Σ_hkl_ Σ_i_ |*I*_hkl,j_ - <*I*_hkl_>| / Σ_hkl_ Σ_i_ *I*_hkl,j_, where *I*_hkl,j_ is the intensity of observation *I*_hkl,j_ and <*I*_hkl_> is the average of symmetry-related observations of a unique reflection.

^c^*R*_cryst_ = Σ||*F*_o_| - |*F*_c_|| / Σ|*F*_o_|, where *F*_o_ and *F*_c_ are observed and calculated structure factor amplitudes, respectively.

^d^*R*_free_ was calculated using a randomly selected 5% of the dataset that was omitted from all stages of refinement.

^e^Ramachandran plots were prepared for all residues other than Gly and Pro.

**
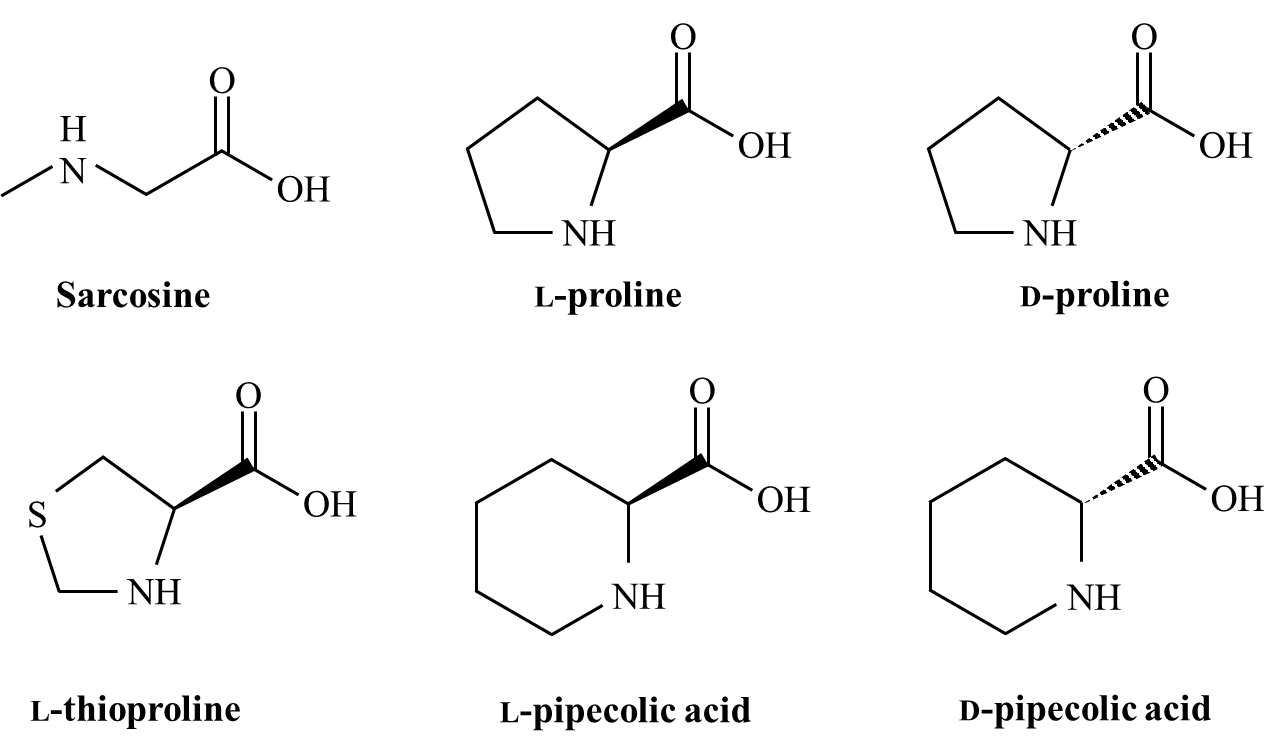
Fig. S1**. Sox substrates.Sarcosine (PubChem CID: 1088), l-proline (PubChem CID: 145742), d-proline (PubChem CID: 8988), l-thioproline (PubChem CID: 93176), l-pipecolic acid (PubChem CID: 439227), d-pipecolic acid (PubChem CID: 736316).

**
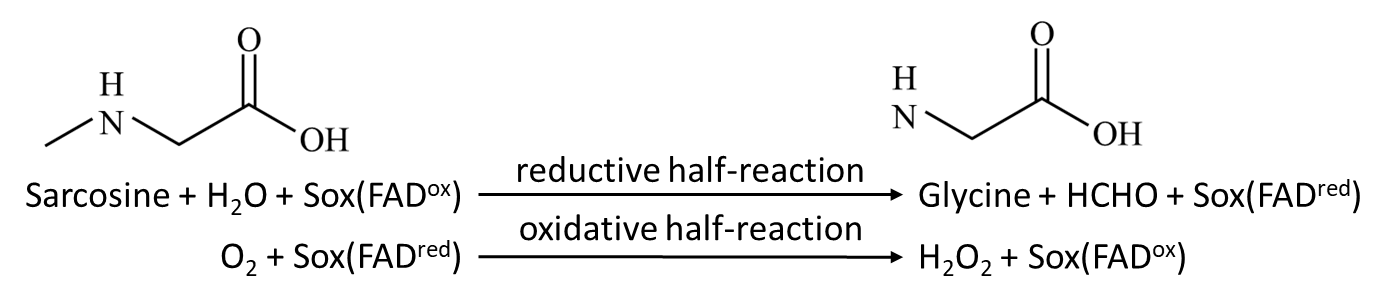
Fig. S2**. Schematic of the Sox reaction.

**
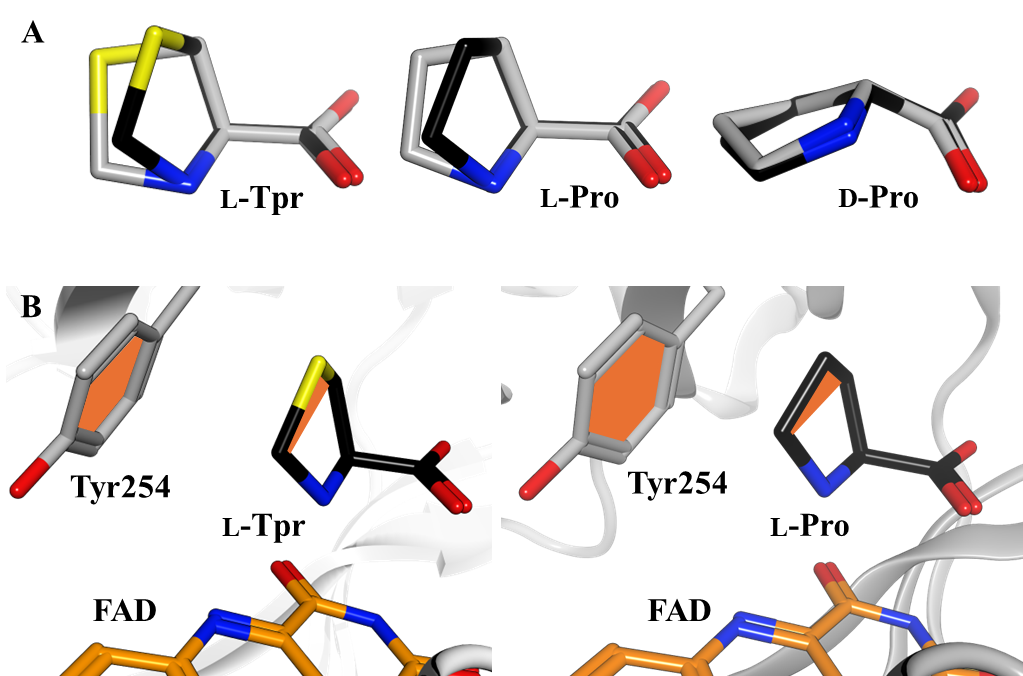
Fig. S3**. Effect of Tyr254 on substrate blinding. (A) Superposition of Sox-bound and free substrate structures. (B) Parallel orientation of five-membered ring imino acids relative to the six-membered ring of Tyr254. The black stick indicates the bound substrate; the gray stick indicates the free substrate. Oxygen is red, nitrogen is blue, and sulfur is yellow. Orange: parallel planes.
